# Antismoking agents do not contribute synergistically to semaglutide’s ability to reduce alcohol intake in rats

**DOI:** 10.3389/fphar.2023.1180512

**Published:** 2023-08-31

**Authors:** Cajsa Aranäs, Sebastian Blid Sköldheden, Elisabet Jerlhag

**Affiliations:** Department of Pharmacology, Institute of Neuroscience and Physiology, The Sahlgrenska Academy at the University of Gothenburg, Gothenburg, Sweden

**Keywords:** GLP-1, alcohol dependence, dopamine, smoking, reward

## Abstract

Preclinical studies have identified glucagon-like peptide-1 receptor (GLP-1R) agonists, and the antismoking agents varenicline and bupropion as tentative agents for treatment of alcohol use disorder (AUD). Combining different medications is a recent approach that has gained attention regarding heterogenous and difficult-to-treat diseases, like AUD. Successfully, this approach has been tested for the combination of varenicline and bupropion as it prevents relapse to alcohol drinking in rats. However, studies assessing the effects of the combination of semaglutide, an FDA-approved GLP-1R agonist for diabetes type II, and varenicline or bupropion to reduce alcohol intake in male and female rats remains to be conducted. Another approach to influence treatment outcome is to combine a medication with feeding interventions like high fat diet (HFD). While HFD reduces alcohol intake, the ability of the combination of HFD and semaglutide to alter alcohol drinking is unknown and thus the subject for a pilot study. Therefore, three intermittent alcohol drinking experiments were conducted to elucidate the effectiveness of these treatment combinations. We show that semaglutide, bupropion or HFD reduces alcohol intake in male as well as female rats. While various studies reveal beneficial effects of combinatorial pharmacotherapies for the treatment of AUD, we herein do not report any additive effects on alcohol intake by adding either varenicline or bupropion to semaglutide treatment. Neither does HFD exposure alter the ability of semaglutide to reduce alcohol intake. Although no additive effects by the combinatorial treatments are found, these findings collectively provide insight into possible monotherapeutical treatments for AUD.

## 1 Introduction

The socioeconomic and medical severity of alcohol use disorder (AUD) is substantial (Rehm et al., 2009), and the limited efficacy of available treatments is thus a major concern (Heilig and Egli, 2006). Novel treatments are therefore needed, and among those that has been identified through preclinical studies are glucagon-like peptide-1 receptor (GLP-1R) agonists, and the antismoking agents varenicline and bupropion (for review see (Chatterjee and Bartlett, 2010; Soderpalm et al., 2020; Tufvesson-Alm et al., 2022).

The gut-brain peptide GLP-1 is foremost known to control glucose homeostasis by its ability to increase pancreatic insulin secretion and reduce hepatic glucose production (Holst and Seino, 2009). GLP-1R agonists are hence approved as treatment of diabetes type II (Nauck et al., 2021). The findings that GLP-1R agonists reduce homeostatic and hedonic feeding and lowers body weight (for review see (Kanoski, 2021), have led to their approval as antiobesity agents (Wilding et al., 2021). Alcohol consumption is another aspect regulated by GLP-1, as acute or repeated administration of various GLP-1R agonists decreases alcohol intake, alcohol seeking and relapse in animals (for review see (Tufvesson-Alm et al., 2022). On a similar note, the GLP-1R agonist exenatide reduces alcohol consumption in overweight AUD patients (Klausen et al., 2022) and polymorphisms of the *GLP-1R* genes is associated with the AUD diagnosis and high alcohol intake (Suchankova et al., 2015). Semaglutide is the most recent clinically available GLP-1R agonist shown to reduce alcohol drinking in rodents (Marty et al., 2020; Aranäs et al., 2023; Chuong et al., 2023).

The smoking cessation agent varenicline is a partial agonist for nicotinic acetylcholine receptors (nAChR) and has been found to reduce alcohol consumption in rats (Steensland et al., 2007; Ericson et al., 2009; Hendrickson et al., 2010; Feduccia et al., 2014; Froehlich et al., 2017). Similarly, varenicline prevents craving for alcohol in alcohol consuming smokers and reduces alcohol intake in patient with AUD (McKee et al., 2009; Mitchell et al., 2012; Litten et al., 2013; de Bejczy et al., 2015; Falk et al., 2015; Verplaetse et al., 2016; Roberts et al., 2017a; Roberts et al., 2017b). Another treatment for smoking is the noradrenaline and dopamine reuptake inhibitor and nAChR antagonist, bupropion (for review see (Gadde and Xiong, 2007; Rigotti et al., 2022). Alike varenicline, bupropion reduces alcohol intake in animal models of AUD (Navarro et al., 2019; Zhou et al., 2019).

To combine different medications is a recent approach that has gained enhanced attention for the treatment of heterogenous diseases. Intriguingly, the manifestation of a disease can be synergistically alleviated when combining medications with distinct neurochemical mechanisms. Successfully, this approach has been tested for the treatment of AUD, where the combination of varenicline and bupropion prevents relapse to alcohol drinking in rats (Soderpalm et al., 2020). Moreover, the treatment of naltrexone together with either varenicline or bupropion reduces alcohol consumption in rats (Zhou et al., 2019; Grodin et al., 2021). The focus of the current study was therefore to assess the effectiveness of the combination of semaglutide and varenicline or bupropion to reduce alcohol intake in male and female rats. Another approach to influence treatment outcome is to combine medications with feeding interventions like high fat diet (HFD) exposure. While HFD reduces alcohol intake (Sirohi et al., 2017a; Sirohi et al., 2017b; Villavasso et al., 2019; Coker et al., 2020), the ability of the combination of HFD and semaglutide to alter alcohol drinking is unknown and thus the subject for a pilot study. Collectively, these alcohol drinking studies in rats of both sexes will contribute towards an unexplored research field where the impact of semaglutide combined with other treatments on alcohol drinking will be elucidated.

## 2 Material and methods

### 2.1 Animals

Male and female Rcc/Han Wistar rats (post-pubertal; Envigo, Horst, Netherland) known to display a high alcohol intake (Simms et al., 2008) were used. They were individually housed in rooms with a temperature of 20°C and 50% humidity. The rats were kept on a reversed light/dark cycle and had free access to regular chow and tap water. The experiments were approved by the Ethics Committee for Animal Experiments, Gothenburg, Sweden and followed the PREPARE and ARRIVE guidelines.

### 2.2 Drugs

Alcohol (95%; Sloveco, Stockholm, Sweden) was diluted in tap water to a final concentration of 20%. Semaglutide (0.026 mg/kg, sc), varenicline (1.5 mg/kg, sc) and bupropion (10 mg/kg, ip) were diluted in saline (0.9% NaCl) and injected 1 hour prior to alcohol exposure. The dose of semaglutide (0.026 mg/kg, sc) was selected as it is lower dose (1/4) than a dose previously shown to reduce alcohol intake in male rats (0.1 mg/kg) (Marty et al., 2020). The doses of varenicline and bupropion were selected because they alone slightly reduce alcohol intake, and when combined together or with naltrexone, they reduce alcohol drinking in rats (Zhou et al., 2019; Soderpalm et al., 2020; Grodin et al., 2021). In the HFD experiment, peanut butter (Green Choice; Coop, Gothenburg, Sweden) was used as HFD. While previous studies have used different designs to decrease alcohol drinking (Sirohi et al., 2017a; Sirohi et al., 2017b; Villavasso et al., 2019; Coker et al., 2020), another design was used as this design previously has been shown to blunt the ability of another GLP-1R agonist to reduce aggressive behaviors (Vestlund et al., 2022).

### 2.3 Intermittent alcohol drinking experiment

Three independent intermittent alcohol drinking experiments were conducted and new rats were used for each experiment. In the intermittent access model, the rats could choose between one alcohol (20%) and one water bottle during three weekly 24-h sessions (Monday, Wednesday, Friday) (Vallof et al., 2020; Kalafateli et al., 2021). For the remaining days two water bottles were available. Bottles were changed when the light was turned off. Throughout the experiment, the 24-h intake of alcohol, water and food as well as body weight were measured. In experiment 1 and 3 the body weight change (difference from the weight the prior day) was also analyzed. After 10 weeks of baseline drinking, the rats were exposed to treatment as described below.

**FIGURE 1:**
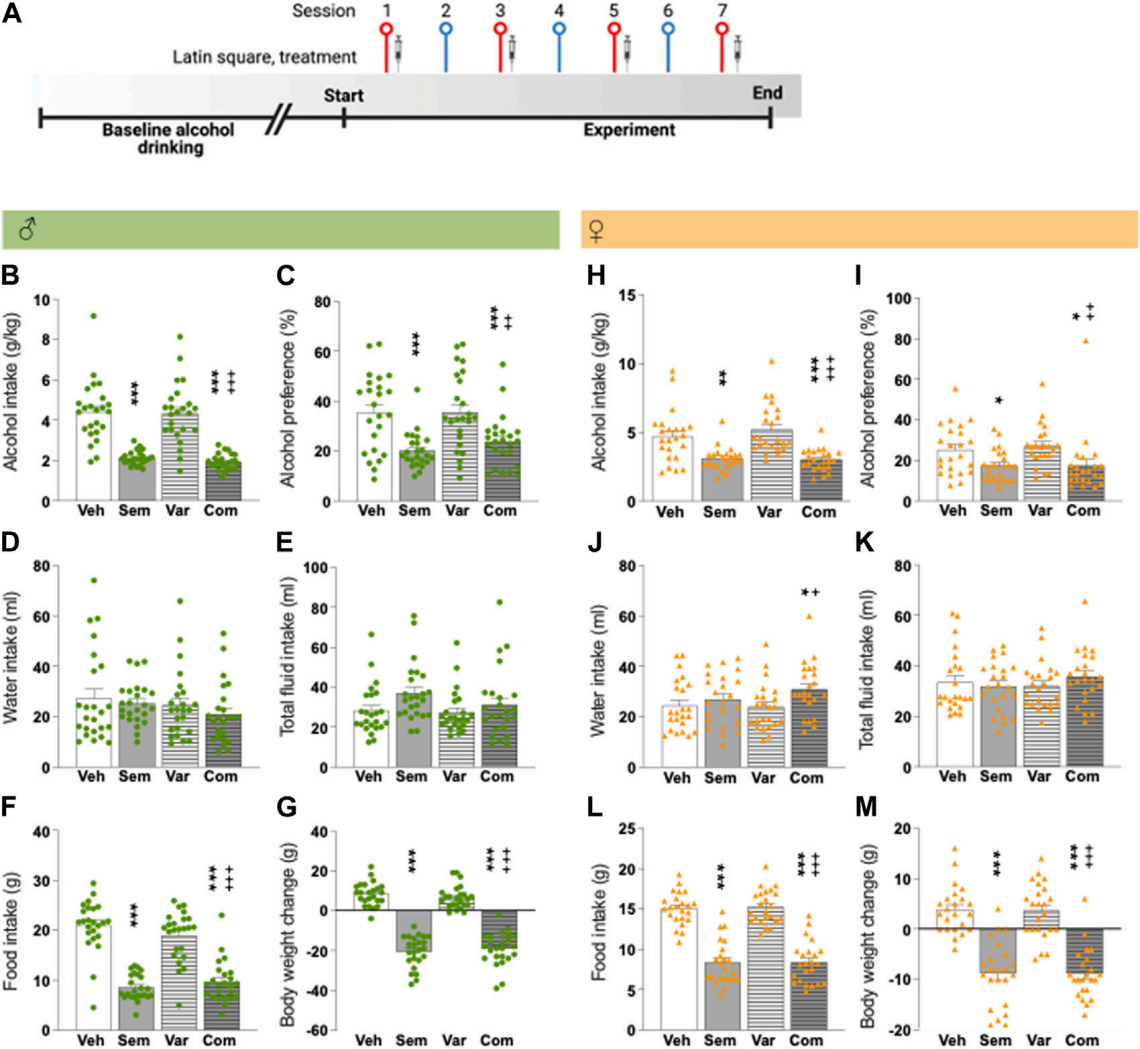
Effects of the combination of semaglutide and varenicline on alcohol intake in male and female rats **(A)** Timeline for the alcohol drinking experiment in which male (*n* = 24) and female (*n* = 23) rats were injected with semaglutide, varenicline or their combination in a Latin square design. Red symbols alcohol drinking session [corresponding to alcohol drinking session shown in **(B–M)**], and blue a water drinking day. The syringe indicates the sessions in which a drug was injected. The timeline was created with BioRender.com. Males: compared to vehicle semaglutide alone or in combination with varenicline decreases **(B)** alcohol intake and **(C)** alcohol preference, but the combination does not decline these alcohol parameters more than semaglutide alone. On other hand, neither treatment altered the **(D)** water and **(E)** total fluid intake. Further, semaglutide as monotherapy or combined with varenicline reduces **(F)** food intake and **(G)** body weight change, but with a similar magnitude. Varenicline does not affect any of the studied parameters, and in comparison to varenicline the combination reduces alcohol intake, preference for alcohol, food intake and body weight change (Figures 1B–G). Females: in comparison to vehicle, semaglutide and the combination decreases **(H)** alcohol intake and **(I)** preference for alcohol, and no additive-like effects were found. Whereas the **(J)** water intake is unaltered by semaglutide or varenicline treatment, their combination increases water intake. On the contrary, **(K)** treatment does not affect the total fluid intake. Further, with a similar magnitude both semaglutide alone or in combination reduces **(L)** food intake and **(M)** body weight change. Varenicline does not affect any of the studied parameters, and in comparison to varenicline the combination reduces alcohol intake, preference for alcohol, food intake and body weight change and increases water intake (Figures 1H–M). Data are presented as mean ± SEM, significant data are illustrated by *p < 0.05, **p < 0.01, ***p < 0.001 in comparison to vehicle. +*p* < 0.05, ++*p* < 0.01, +++*p* < 0.001 in comparison to varenicline. Vehicle (Veh), semaglutide (Sem), varenicline (Var), semaglutide and varenicline combination (Com).

The first alcohol drinking experiment explores the effect of semaglutide (0.026 mg/kg, sc) combined with varenicline (1.5 mg/kg, sc) on alcohol consumption. After baseline drinking, male (*n* = 24) and female rats (*n* = 23) were acutely injected with i) semaglutide and varenicline, ii) vehicle and semaglutide, iii) varenicline and vehicle or iv) double vehicle injections in a 4x4 Latin Square design. The four treatment sessions (Monday, Wednesday, Friday, Sunday) were separated by one water drinking session, and each rat was injected with all treatments in a stratified manner (see Figure 1A for timeline). A Latin squared design was used to reduce the number of animals used.

**FIGURE 2:**
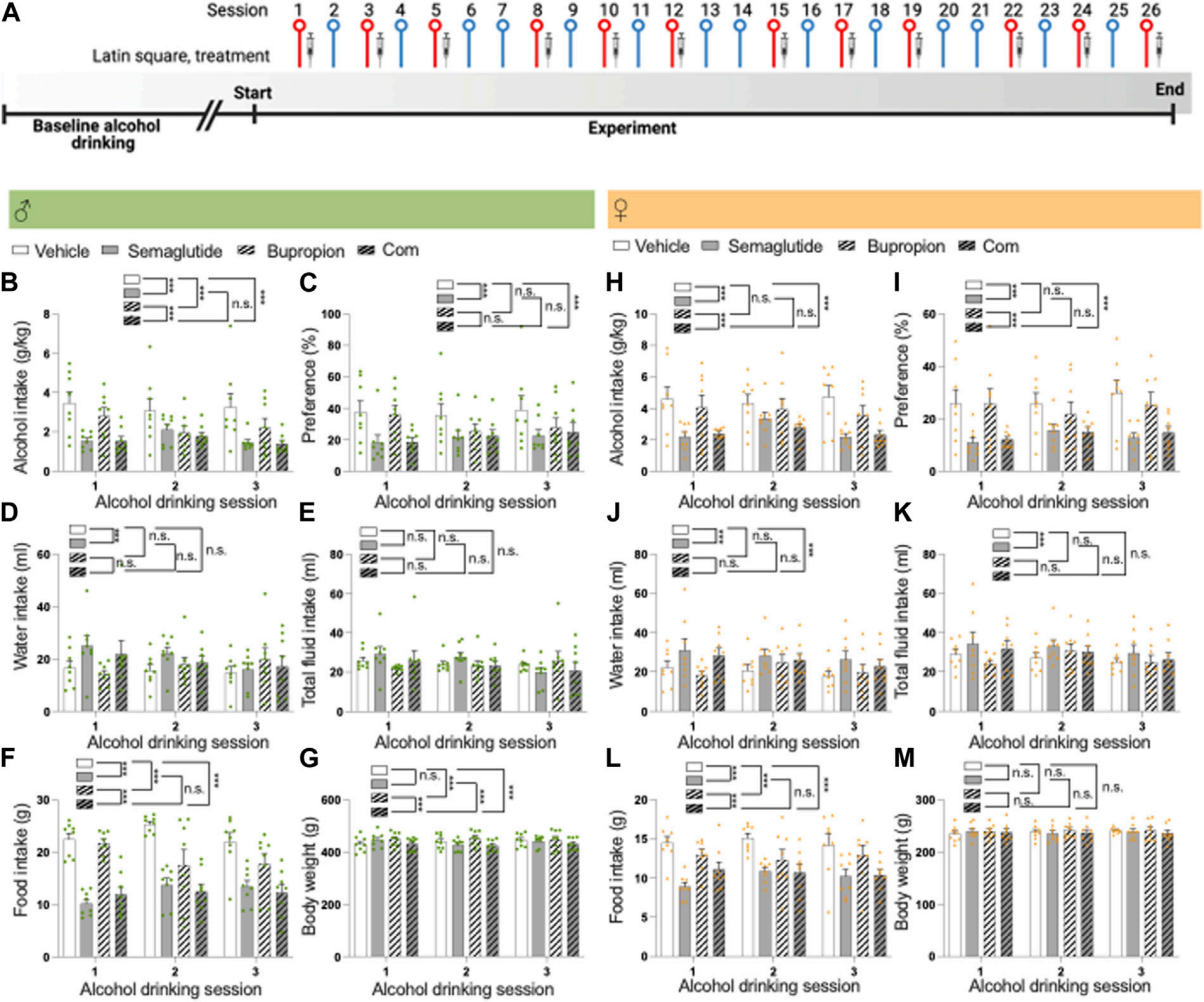
Effects of the combination of semaglutide and bupropion on alcohol intake in male and female rats **(A)** Timeline for the alcohol drinking experiment in which male (*n* = 8) and female (*n* = 8) rats were injected with semaglutide, bupropion or their combination in a Latin square design (three injections for each treatment). Red symbols alcohol drinking session [corresponding to alcohol drinking sessions 1–3 in **(B–M)**], and blue a water drinking day. The syringe indicates the sessions in which a drug was injected. The timeline was created with BioRender.com. Males: Compared to vehicle, semaglutide alone or in combination with bupropion decreases **(B)** alcohol intake and **(C)** preference for alcohol, and the decline is similar between these groups. Compared to bupropion monotherapy, the combination treatment lowers alcohol intake, but not preference. Moreover, in comparison to vehicle bupropion reduces alcohol intake without altering preference for alcohol. Although semaglutide **(D)** increases water intake, **(E)** the total fluid intake was unaltered by treatment. **(F)** Compared to vehicle, semaglutide alone or in combination with bupropion decreases food intake, and the decline is similar between these groups. In comparison to vehicle, bupropion reduces food intake. Compared to the bupropion monotherapy, the combination treatment lowers food intake further. **(G)** When it comes to the body weight, bupropion increases this parameter whereas its unaltered by semaglutide. The body weight is lower in rats treated with the combination therapy compared to either monotherapy. Females: Semaglutide, alone or in combination, reduces **(H)** alcohol intake and **(I)** preference for alcohol compared to vehicle, and this decrease is similar between the semaglutide and combination groups. Bupropion does not influence these alcohol parameters in females. The alcohol intake and preference are lower in female rats treated with the combination compared to those treated with bupropion. Whereas both semaglutide and the combination increase **(J)** water intake, only **(K)** semaglutide enhances the total fluid intake. **(L)** While semaglutide, bupropion and the combination reduce food intake with a similar magnitude, the combination treatment decreases it further compared to bupropion. **(M)** There are no treatment effects on body weight. Data are presented as mean ± SEM, significant data are illustrated by ***p < 0.001. Vehicle (Veh), semaglutide (Sem), bupropion (Bup), semaglutide and bupropion combination (Com).

The second alcohol drinking experiment used a repeated 4x4 Latin square design with repeated injections. Male (*n* = 8) and female rats (*n* = 8) were injected with i) semaglutide (0.026 mg/kg) and bupropion (10 mg/kg), ii) vehicle and semaglutide, iii) bupropion and vehicle, or iv) two vehicle injections at the three weekly drinking days (Monday, Wednesday, Friday). These three injections per week occurred for four subsequent weeks. Each week was separated by two water drinking days (see Figure 2A for timeline). This Latin square design ensured that each rat got all treatments and was used to reduce the number of animals used.

**FIGURE 3:**
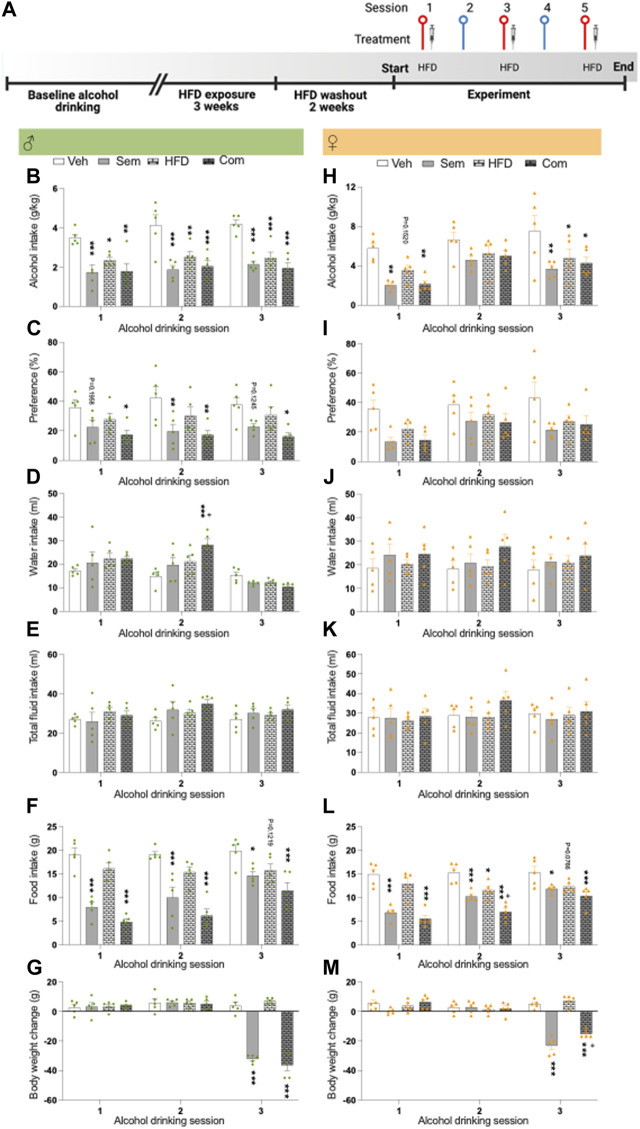
Effects of high fat diet and its combination with semaglutide on alcohol drinking in male and female rats **(A)** Timeline for the alcohol drinking experiment in which male (*n* = 20) and female (*n* = 20) rats were exposed to high fat diet for 3 weeks. After 2 weeks of washout from HFD, rats were injected with semaglutide or vehicle, and exposed to HFD or regular chow. Red symbols alcohol drinking session [corresponding to alcohol drinking sessions 1–3 in **(B–M)**], and blue a water drinking day. The syringe indicates the sessions in which a drug was injected and HFD indicates when this was exposed. The timeline was created with BioRender.com. Males: **(B)** The alcohol intake is reduced by semaglutide (Sem), high fat diet (HFD) or their combination (Com), and the semaglutide response is unaltered by HFD. **(C)** Compared to vehicle (Veh), both semaglutide and the combination decrease the preference for alcohol. This decrease was similar in both these groups. **(D)** While the combination treatment increase water intake, **(E)** the total fluid intake is not affected by treatments. Semaglutide as well as the combination treatment decreases **(F)** food intake and **(G)** the body weight change. Moreover, HFD tends to decrease food intake and also tends to additively lower the ability of semaglutide to reduce food intake. Females: **(H)** both semaglutide treatment alone and its combination with HFD reduce alcohol drinking. Moreover, HFD decreases alcohol intake, while it does not alter the ability of semaglutide to lower alcohol intake. neither treatment affects **(I)** preference for alcohol, **(J)** water intake or **(K)** total fluid intake. **(L)** HFD, semaglutide or their combination reduces food intake, and HFD further reduces the ability of semaglutide to reduce food intake. **(M)** The body weight change is reduced after treatment with either semaglutide or the combination. HFD blunts the ability of semaglutide to reduce body weight change. Data are presented as mean ± SEM, significant data are illustrated by, *p < 0.05, **p < 0.01, ***p < 0.001 when compared to vehicle. +*p* < 0.05 illustrates significant effect when comparing the combination treatment to HFD.

The first part of the third alcohol drinking experiment aimed to confirm previous studies in which exposure to HFD reduces alcohol drinking in male rats (Sirohi et al., 2017a; Sirohi et al., 2017b; Villavasso et al., 2019; Coker et al., 2020) and to confirm a similar outcome in female rats. After baseline drinking, male (*n* = 20) and female (*n* = 20) rats had access to HFD 4 hours every weekday (Monday-Friday) for 3 weeks. The exposure to HFD was timed with the exposure to alcohol, as we wanted the rats to choose between alcohol and HFD rather being full from HFD intake. The second part of this alcohol drinking experiment explored the influence of the combination of HFD and semaglutide on alcohol intake in rats of both sexes. Therefore, the rats did not have access to HFD for 2 weeks. After this 2-week HFDwash-out, the rats were treated with semaglutide (0.026 mg/kg) or vehicle at three alcohol drinking sessions (Monday, Wednesday, Friday). Additionally, the rats were during these days exposed to HFD for 2 hours or chow (timed with the initial exposure to alcohol): creating four treatment groups (vehicle-chow, vehicle-HFD, semaglutide-chow, and semaglutide-HFD) (see Figure 3A for timeline).

### 2.4 Statistical analysis

The first data set was analyzed with a repeated measures one-way ANOVA with a Tukey’s post-hoc test. A statistician at University of Gothenburg analyzed the second data set with a Mixed Model Analysis of Variance. Data from the third test were analyzed with a repeated two-way ANOVA with Tukey’s post-hoc test. The probability of *p* < 0.05 is considered as statistically significant and post-hoc test were adjusted for multiple testing.

## 3 Results

### 3.1 Effects of the semaglutide and varenicline combination on alcohol intake in male and female rats

In male rats, alcohol drinking during baseline was 4.6 **±** 0.3 g/kg. Compared to vehicle, semaglutide alone or in combination with varenicline decreased alcohol intake and alcohol preference, but the combination did not suppress these alcohol parameters more than semaglutide alone (Figures 1B, C; Table 1). On the other hand, neither treatment altered the water and total fluid intake (Figures 1D, E; Table 1). Further, semaglutide as monotherapy or combined with varenicline reduced food intake and body weight change, but with a similar magnitude (Figures 1F, G; Table 1). Varenicline did not affect any of the studied parameters, and compared to varenicline the combination reduced alcohol intake, preference for alcohol, food intake and body weight change (Figures 1B–G). Each treatment included 24 male rats, and no animals were excluded in the present experiment.

**TABLE 1:** Statistical summary of the effects of the semaglutide and varenicline combination on measurements in male and female rats. Vehicle (Veh), semaglutide (sem), varenicline (Var), semaglutide and varenicline in combination (Com).

Treatment effects after the combination of semaglutide and varenicline							
*Sex*	*Measurements*	*Statistical analysis*	*Veh*vs.*Sem*	*Veh*vs.*Var*	*Veh*vs.*Com*	*Var*vs.*Com*	*Sem*vs.*Com*
Males	Alcohol intake	F(3,69) = 39.90, *p* < 0.0001	*p* < 0.0001	*p* = 0.9941	*p* < 0.0001	*p* < 0.0001	*p* = 0.9331
	Preference for alcohol	F(3,69) = 12.87, *p* < 0.0001	*p* < 0.0001	*p* > 0.9999	*p* = 0.0025	*p* = 0.0020	*p* = 0.6356
	Water intake	F(3,69) = 1.01, *p* = 0.3947	—				
	Total fluid intake	F(3,69) = 2.42, *p* = 0.0739	—				
	Food intake	F(3,69) = 52.84, *p* < 0.0001	*p* < 0.0001	*p* = 0.3813	*p* < 0.0001	*p* < 0.0001	*p* = 0.8214
	Body weight change	F(3,69) = 193.50, *p* < 0.0001	*p* < 0.0001	*p* = 0.7284	*p* < 0.0001	*p* < 0.0001	*p* = 0.7871
Females	Alcohol intake	F(3,66) = 14.72, *p* < 0.0001	*p* = 0.0012	*p* = 0.6040	*p* = 0.0007	*p* < 0.0001	*p* = 0.9990
	Preference for alcohol	F(3,66) = 7.44, *p* = 0.0002	*p* = 0.0239	*p* = 0.8877	*p* = 0.0221	*p* = 0.0029	*p* > 0.9999
	Water intake	F(3,66) = 3.66, *p* = 0.0168	*p* = 0.7146	*p* = 0.9959	*p* = 0.0386	0.0214	*p* = 0.3430
	Total fluid intake	F(3,66) = 0.65, *p* = 0.5874	—				
	Food intake	F(3,66) = 72.02, *p* < 0.0001	*p* < 0.0001	*p* = 0.9951	*p* < 0.0001	*p* < 0.0001	*p* > 0.9999
	Body weight change	F(3,66) = 44.33, *p* < 0.0001	*p* < 0.0001	*p* = 0.9990	*p* < 0.0001	*p* < 0.0001	*p* > 0.9999

The alcohol intake was 4.4 ± 0.3 g/kg for female rats during baseline. In comparison to vehicle, semaglutide and the combination decreased alcohol intake and preference for alcohol, and no like effects were found (Figures 1H, I). Whereas the water intake was unaltered by semaglutide or varenicline treatment, their combination increased water intake (Figure 1J). On the contrary, treatment did not affect the total fluid intake (Figure 1K). Further, with a similar magnitude both semaglutide alone or in combination reduced food intake and body weight (Figures 1L, M). Varenicline did not affect any of the studied parameters, and in comparison to varenicline, the combination reduced alcohol intake, preference for alcohol, food intake and body weight and increased water intake (Figures 1H–M). Each treatment included 23 female rats, and no animals were excluded in the present experiment.

As shown before, the water intake (when adjusting for body weight) was higher in female compared to male rats (63.5 ± 8.4 mL/kg for males and 173.0 ± 10.0 mL/kg for females, t(45) = 2.98, *p* = 0.0046). For both female and male rats, treatment reduced the body weight change whereas this reduction was not evident when comparing the actual weight of the rats (data not shown). The alcohol intake of rats treated with the vehicle in the session after drug treatment was comparable to their baseline drinking levels (data not shown), indicating the absence of any remaining inhibitory effect from the prior treatment.

### 3.2 Effects of the combination of semaglutide and bupropion on consumption patterns in male and female rats

In males, the baseline alcohol intake prior to treatment was 3.8 ± 0.2 g/kg. Compared to vehicle, semaglutide alone or in combination with bupropion decreased alcohol intake and preference for alcohol, and the decline was similar between these groups (Figures 2B, C; Table 2). Compared to bupropion monotherapy, the combination treatment lowered alcohol intake, but not preference. Moreover, in comparison to vehicle, bupropion reduced alcohol intake without altering preference for alcohol. Although semaglutide increased water intake, the total fluid intake was unaltered by treatment (Figures 2D, E). Compared to vehicle, semaglutide alone or in combination with bupropion decreased food intake, and the decline was similar between these groups (Figure 2F). In comparison to vehicle, bupropion reduced food intake. Compared to the bupropion monotherapy, the combination treatment lowered food intake further. When it comes to the body weight, bupropion increased this parameter whereas it was unaltered by semaglutide (Figure 2G). The body weight was lower in rats treated with the combination therapy compared to either monotherapy or vehicle. Each treatment included 8 male rats, and no animals were excluded in the present experiments.

**TABLE 2:** Statistical summary of the effects of the semaglutide and bupropion combination on measurements in male and female rats. Vehicle (Veh), semaglutide (Sem), bupropion (Bup), semaglutide and bupropion in combination (Com).

Treatment effects after the combination of semaglutide and bupropion							
*Sex*	*Measurements*	*Statistical analysis*	*Veh* vs.*Sem*	*Veh*vs.*Bup*	*Veh*vs.*Com*	*Bup*vs.*Com*	*Sem*vs.*Com*
Males	Alcohol intake	F(15,95) = 9.07, *p* < 0.0001	*p* < 0.0001	*p* < 0.0001	*p* < 0.0001	*p* < 0.0001	*p* > 0.05
	Preference for alcohol	F(15,95) = 9.68, *p* < 0.0001	*p* < 0.0001	*p* > 0.05	*p* < 0.0001	*p* > 0.05	*p* > 0.05
	Water intake	F(15,95) = 5.90, *p* < 0.0001	—				
	Total fluid intake	F(15,95) = 4.75, *p* < 0.0001	—				
	Food intake	F(15,95) = 12.50, *p* < 0.0001	*p* < 0.0001	*p* < 0.0001	*p* < 0.0001	*p* < 0.0001	*p* > 0.05
	Body weight	F(15,95) = 6.37, *p* < 0.0001	*p* > 0.05	*p* < 0.0001	*p* < 0.0001	*p* < 0.0001	*p* < 0.0001
Females	Alcohol intake	F(15,95) = 10.97, *p* < 0.0001	*p* < 0.0001	*p* > 0.05	*p* < 0.0001	*p* < 0.0001	*p* > 0.05
	Preference for alcohol	F(15,95) = 15.34, *p* < 0.0001	*p* < 0.0001	*p* > 0.05	*p* < 0.0001	*p* < 0.0001	*p* > 0.05
	Water intake	F(15,95) = 11.56, *p* < 0.0001	*p* < 0.0001	*p* > 0.05	*p* < 0.0001	*p* > 0.05	*p* > 0.05
	Total fluid intake	F(15,95) = 8.09, *p* < 0.0001	*p* < 0.0001	*p* > 0.05	*p* > 0.05	*p* > 0.05	*p* > 0.05
	Food intake	F(15,95) = 6.56, *p* < 0.0001	*p* < 0.0001	*p* < 0.001	*p* < 0.0001	*p* < 0.0001	*p* > 0.05
	Body weight	F(15,95) = 1.40, *p* = 0.2490	—				

During the baseline the alcohol intake was 6.1 ± 0.2 g/kg for female rats. Semaglutide alone or in combination reduced alcohol intake and preference for alcohol compared to vehicle, and this decrease was similar between these groups (Figures 2H, I; Table 2). In contrast to males, bupropion did not influence these alcohol parameters in females. The alcohol intake and preference were lower in female rats treated with the combination compared to those treated with bupropion. Whereas both semaglutide and the combination increased water intake, only semaglutide enhanced the total fluid intake (Figures 2J, K). Semaglutide, bupropion and the combination reduced food intake without changing the body weight (Figures 2L, M). The combination treatment reduced food intake with a similar magnitude to semaglutide, whereas it decreased it further compared to bupropion. Each treatment included 8 female rats, and no animals were excluded in the present experiments.

For both female and male rats, treatment affected the body weight whereas this change was not evident when comparing the body weight change of the rats (data not shown). The alcohol intake of rats treated with the vehicle in the session after drug treatment was comparable to their baseline drinking levels (data not shown), indicating the absence of any remaining inhibitory effect from the prior treatment.

### 3.3 Effects of high fat diet and its combination with semaglutide on alcohol drinking in male and female rats

As shown before, exposure to HFD reduced alcohol intake in male rats (baseline drinking: 4.2 **±** 0.3, HFD exposure 2.7 **±** 0.2: t(38) = 4.12, *p* = 0.0002). We further show that HFD exposure decreased alcohol drinking in female rats (baseline drinking: 5.4 **±** 0.4, HFD exposure 3.9 **±** 0.3: t(38) = 3.13, *p* = 0.0034).

After 2 weeks of washout from HFD, the interaction between HFD and semaglutide on alcohol drinking was tested. The alcohol intake was similar (F(3,16) = 0.00, *p* > 0.9999) between male rats later assigned to vehicle (vehicle-chow; 3.1 ± 0.3), semaglutide (semaglutide-chow; 3.1 ± 0.4), HFD (vehicle-HFD; 3.1 ± 0.8), combination treatment (semaglutide-HFD; 3.1 ± 0.5). In male rats, the alcohol intake was reduced by HFD, semaglutide or their combination, and the semaglutide response was unaltered by HFD (Figure 3B; Table 3). Both semaglutide and the combination decreased the preference for alcohol (Figure 3C). While the combination treatment increased water intake compared to vehicle or semaglutide, the total fluid intake was not affected by treatments (Figures 3D, E). Food intake and body weight difference was lower in male rats treated with semaglutide and the combination treatment (Figures 3F, G). Moreover, HFD tended to decrease food intake and also tended to additively lower the ability of semaglutide to reduce food intake. Each treatment included 20 male rats, and no animals were excluded in the present experiments.

**TABLE 3:** Statistical summary of the effects of the semaglutide and high fat diet combination on alcohol intake in male and female rats. Vehicle (Veh), semaglutide (Sem), high fat diet (HFD), semaglutide and high fat diet in combination (Com).

*Sex*	*Measurements*	*Statistical analysis*		*Veh*vs.*Sem*	*Veh*vs.*HFD*	*Veh*vs.*Com*	*HFD*vs.*Com*	*Sem*vs.*Com*
Males	Alcohol intake	Treatment	F(3,16) = 20.83, *p* < 0.0001	S1: *p* = 0.0007	S1: *p* = 0.0437	S1: *p* = 0.0010	S1: *p* = 5472	S1: *p* = 0.9993
		Time	F(2,32) = 2.13,*p* = 0.1354	S2: *p* < 0.0001	S2: *p* = 0.0022	S2: *p* < 0.0001	S2: *p* = 0.6484	S2: *p* = 0.9764
		Interaction	F(6,32) = 0.38, *p* = 0.8892	S3: *p* < 0.0001	S3: *p* = 0.0009	S3: *p* < 0.0001	S3: *p* = 0.5949	S2: *p* = 0.9695
	Alcohol preference	Treatment	F(3,16) = 5.24, *p* = 0.0104	S1: *p* = 0.1958	S1: *p* = 0.6120	S1: *p* = 0.0371	S1: *p* = 0.4130	S1: *p* = 0.8689
		Time	F(2,32) = 0.33, *p* = 0.7213	S2: *p* = 0.0069	S2: *p* = 0.2797	S2: *p* = 0.0023	S2: *p* = 0.2099	S2: *p* = 0.9814
		Interaction	F(6,32) = 0.69, *p* = 0.6574	S3: *p* = 0.1245	S3: *p* = 0.7215	S3: *p* = 0.0115	S3: *p* = 0.1420	S2: *p* = 0.7591
	Water intake	Treatment	F(3,16) = 2.08, *p* = 0.1440	S1: *p* = 0.6537	S1: *p* = 0.3007	S1: *p* = 0.3279	S1: *p* > 0.9999	S1: *p* = 0.9455
		Time	F(2,32) = 22.21, *p* < 0.0001	S2: *p* = 0.3872	S2: *p* = 0.1690	S2: *p* = 0.0003	S2: *p* = 0.1054	S2: *p* = 0.0337
		Interaction	F(6,32) = 3.59, *p* = 0.0078	S3: *p* = 0.6537	S3: *p* = 0.7494	S3: *p* = 0.3582	S3: *p* = 0.9136	S3: *p* = 0.9601
	Total fluid intake	Treatment	F(3,16) = 0.99, *p* = 0.4236	—				
		Time	F(2,32) = 2.71, *p* = 0.0821					
		Interaction	F(6,32) = 1.73, *p* = 0.1472					
	Food intake	Treatment	F(3,16) = 26.99, *p* < 0.0001	S1: *p* < 0.0001	S1: *p* = 0.3675	S1: *p* < 0.0001	S1: *p* < 0.0001	S1: *p* = 0.3331
		Time	F(2,32) = 13.40, *p* < 0.0001	S2: *p* < 0.0001	S2: *p* = 0.1872	S2: *p* < 0.0001	S2: *p* < 0.0001	S2: *p* = 0.1725
		Interaction	F(6,32) = 3,77, *p* = 0.0059	S3: *p* = 0.0315	S3: *p* = 0.1219	S3: *p* = 0.0002	S3: *p* = 0.1015	S3: *p* = 0.3059
	Body weight change	Treatment	F(3,16) = 67.92, *p* < 0.0001	S1: *p* = 0.9967	S1: *p* = 0.9922	S1: *p* = 0.9433	S1: *p* = 0.9922	S1: *p* = 0.9851
		Time	F(2,32) = 100.10,*p* < 0.0001	S2: *p* = 0.9999	S2: *p* > 0.9999	S2: *p* = 0.9967	S2: *p* = 0.9967	S2: *p* = 0.9922
		Interaction	F(6,32) = 36.91, *p* < 0.0001	S3: *p* < 0.0001	S3: *p* = 0.6790	S3: *p* < 0.0001	S3: *p* < 0.0001	S3: *p* = 0.4630
Females	Alcohol intake	Treatment	F(3,16) = 5.44, *p* = 0.0090	S1: *p* = 0.0033	S1: *p* = 0.1520	S1: *p* = 0.0047	S1: *p* = 0.5056	S1: *p* = 0.9994
		Time	F(2,32) = 21.72, *p* < 0.0001	S2: *p* = 0.1917	S2: *p* = 0.5419	S2: *p* = 0.3819	S2: *p* = 0.9930	S2: *p* = 0.9752
		Interaction	F(6,32) = 1.23, *p* = 0.3194	S3: *p* = 0.0026	S3: *p* = 0.0480	S3: *p* = 0.0140	S3: *p* = 0.9627	S3: *p* = 0.4630
	Alcohol preference	Treatment	F(3,16) = 2.52, *p* = 0.0952					
		Time	F(2,32) = 14.96, *p* < 0.0001					
		Interaction	F(6,32) = 1.26, *p* = 0.3057					
	Water intake	Treatment	F(3,16) = 0.73, *p* = 0.5502	—				
		Time	F(2,32) = 0.33, *p* = 0.7231					
		Interaction	F(6,32) = 0.87, *p* = 0.5296					
	Total fluid intake	Treatment	F(3,16) = 0.40, *p* = 0.7553	—				
		Time	F(2,32) = 2.22, *p* = 0.1255					
		Interaction	F(6,32) = 1.09, *p* = 0.3902					
	Food intake	Treatment	F(3,16) = 20.46, *p* < 0.0001	S1: *p* < 0.0001	S1: *p* = 0.3785	S1: *p* < 0.0001	S1: *p* < 0.0001	S1: *p* = 0.7074
		Time	F(2,32) = 18.78, *p* < 0.0001	S2: *p* = 0.0007	S2: *p* = 0.0203	S2: *p* < 0.0001	S2: *p* = 0.0020	S2: *p* = 0.0466
		Interaction	F(6,32) = 7.88, *p* < 0.0001	S3: *p* = 0.0298	S3: *p* = 0.0766	S3: *p* = 0.0009	S3: *p* = 0.3785	S3: *p* = 0.6164
	Body weight change	Treatment	F(3,16) = 48.85, *p* < 0.0001	S1: *p* = 0.1535	S1: *p* = 0.8906	S1: *p* = 0.9887	S1: *p* = 0.7310	S1: *p* = 0.0788
		Time	F(2,32) = 32.72, *p* < 0.0001	S2: *p* = 0.9998	S2: *p* = 0.9638	S2: *p* = 0.9952	S2: *p* = 0.9952	S2: *p* = 0.9887
		Interaction	F(6,32) = 19.10, *p* < 0.0001	S3: *p* < 0.0001	S3: *p* = 0.7761	S3: *p* < 0.0001	S3: *p* < 0.0001	S3: *p* = 0.0133

In female rats later assigned to vehicle (vehicle-chow; 4.4 ± 0.9), semaglutide (semaglutide-chow; 4.4 ± 0.8), HFD (vehicle-HFD; 4.4 ± 0.6), combination treatment (semaglutide-HFD; 4.4 ± 1.3) the baseline alcohol intake was similar (F(3,16) = 0.00, *p* > 0.9999). In these alcohol drinking female rats, semaglutide, HFD and their combination reduced alcohol drinking (Figure 3H; Table 3). Moreover, HFD did not alter the ability of semaglutide to reduce alcohol intake. Neither treatment affected preference for alcohol, water intake or total fluid intake (Figures 3I–K). The food intake was reduced by semaglutide, HFD and their combination, and the semaglutide response was additively reduced by HFD (Figure 3L). The body weight change was reduced after treatment with either semaglutide or the combination, but was unaffected by HFD (Figure 3M). In contrast to the potential of HFD to enhance the ability of semaglutide to reduce food intake, HFD blunts the ability of semaglutide to reduce body weight. Each treatment included 20 female rats, and no animals were excluded in the present experiments.

For both female and male rats, treatment reduced the body weight change whereas this reduction was not evident when comparing the actual weight of the rats (data not shown).

## 4 Discussion

Supporting previous literature, we here show that semaglutide, bupropion or HFD reduces alcohol intake in male rats. We further extend these findings as we show that either of these monotherapies decrease alcohol drinking in female rats. While various studies reveal beneficial effects of combinatorial pharmacotherapies for the treatment of AUD, we herein do not report any additive effects on alcohol intake by adding either varenicline or bupropion to semaglutide treatment. Neither does HFD exposure alter the ability of semaglutide to reduce alcohol intake. These findings collectively provide insight into possible treatments for AUD.

In each of the present alcohol drinking experiments, acute or repeated semaglutide injections reduce alcohol intake and preference for alcohol in both male and female rats. These findings confirm a previous study in which an acute injection of a higher semaglutide dose (0.1 mg/kg) decreases alcohol intake in male rats (Marty et al., 2020). They further support the recent studies revealing that acute and repeated semaglutide treatment, at similar doses, reduces alcohol intake, relapse drinking and binge drinking in male and female rodents (Aranäs et al., 2023; Chuong et al., 2023). They are in further accordance with studies on other GLP-1R agonists, where their systemic administration lowers alcohol intake (Sanchis-Segura and Spanagel, 2006; Davis et al., 2012; Egecioglu et al., 2013; Shirazi et al., 2013; Suchankova et al., 2015; Vallof et al., 2015; Thomsen et al., 2017; Thomsen et al., 2019; Marty et al., 2020; Vallof et al., 2020). Besides, these findings are supported by human genetics studies in which polymorphisms of the *GLP-1R* gene are associated with high alcohol intake as well as AUD (Suchankova et al., 2015). While semaglutide has not been tested in patients with AUD, the GLP-1R agonist exenatide decreases alcohol consumption in overweight patients with AUD (Klausen et al., 2022). As central rather than peripheral GLP-1R appear to control alcohol intake (Sirohi et al., 2016), brain regions central for semaglutide’s ability to reduce alcohol intake remains to be determined. Among areas of interest is those central for reward such as nucleus accumbens, ventral tegmental area and laterodorsal tegmental area as they participate in the interaction between alcohol and GLP-1 (Shirazi et al., 2013; Abtahi et al., 2018; Vallöf et al., 2019; Colvin et al., 2020; Dixon et al., 2020). Additionally, semaglutide is detected in nucleus accumbens after its systemic injection in alcohol drinking male and female rats (Aranäs et al., 2023). Peripheral mechanisms may also contribute as semaglutide mainly penetrates circumventricular organs and that its brain distribution is limited, at least in alcohol-naive rats (Gabery et al., 2020).

While previous studies in male rats report that varenicline decreases alcohol drinking (Steensland et al., 2007; Ericson et al., 2009; Hendrickson et al., 2010; Feduccia et al., 2014; Froehlich et al., 2017), this was not confirmed in the present study of male and female rats using a similar dose of varenicline. Support of our data is provided by a recent study in which varenicline as monotherapy does not decrease alcohol intake in male rats (Soderpalm et al., 2020). The rationale for this discrepancy remains to be established, but may lay in differences in baseline drinking or in time of alcohol exposure prior to treatment. For instance, varenicline displayed a robust decline in rats that consumed high amounts of alcohol for 12 weeks prior to treatment (Steensland et al., 2012). Moreover, varenicline appears to have a robust effect on alcohol intake when rats have been deprived of alcohol (Froehlich et al., 2017; Soderpalm et al., 2020), indicating that the treatment outcome also depends on abstinence exposure. Other tentative factors are strain of rats and route of administration. Indeed, as opposed to subcutaneous injections to outbred rats as herein, oral administration of varenicline to alcohol preferring rats reduces alcohol intake (Froehlich et al., 2017; Czachowski et al., 2018). Moreover, as human studies reveal effects of varenicline in smokers with AUD (McKee et al., Mitchell et al., 2012; Litten et al., 2013; Roberts et al., 2017b; Roberts et al., 2017a; Falk et al., 2015; de Bejczy et al., 2015; Verplaetse et al., 2016), the possibility that varenicline reduces alcohol drinking in nicotine exposed rats should be considered. We further show that bupropion as monotherapy decreases alcohol drinking in male rats, confirming previous studies (Navarro et al., 2019; Zhou et al., 2019). These findings are extended as we reveal that bupropion also lowers alcohol intake in female rats. It should however be noted that neither varenicline nor bupropion adds towards semaglutides ability to decrease alcohol intake. This raises the possibility that the downstream mechanisms targeted by these agents do not interact and thus do not provide any additive effects on alcohol drinking. Although negative findings that are in contrast to other combinatorial pharmacotherapy that additively reduces alcohol drinking (Zhou et al., 2019; Soderpalm et al., 2020; Grodin et al., 2021), they contribute towards an increased knowledge regarding which combinations could have beneficial effects.

In the current study, we selected doses that were intended to have a minimal impact on reducing alcohol intake. It was however evident that semaglutide profoundly reduces alcohol drinking and that varenicline did not reduce alcohol intake alone. As combination of other doses might have a different treatment outcome, the lack of combinations of higher/lower doses should therefore be considered as a limitation. Moreover, as semaglutide possesses a robust decrease in alcohol intake alone, the possibility that treatment combination cannot lower the intake further should be considered. However, other medications like dulaglutide can reduce alcohol more than seen herein (Vallof et al., 2020).

As shown before (Sirohi et al., 2017a; Sirohi et al., 2017b; Villavasso et al., 2019; Coker et al., 2020), HFD reduces alcohol drinking in male rats. Moreover, we found a similar outcome in female rats. On the other hand, HFD does not influence the ability of semaglutide to reduce alcohol intake in neither male nor female alcohol exposed rats. A finding in contrast to other behaviors in which HFD blunts the treatment outcome of GLP-1R agonists (Williams et al., 2011; Duca et al., 2013; Mella et al., 2017; Williams et al., 2018; Al Helaili et al., 2020; Vestlund et al., 2022). Specifically, a similar HFD design blunts the ability of exendine-4, another GLP-1R agonist, to reduce aggression (Vestlund et al., 2022). Furthermore, since we have observed a significant decrease in alcohol consumption following HFD exposure, further investigations are needed to determine whether there is an additive effect between adjusted HFD designs and semaglutide.

In accordance with previous studies in obese/diabetic man or rodents (Martins et al., 2022; O'Neil et al., 2018; Wilding et al., 2021; Gabery et al., 2020), semaglutide decreases food intake in the alcohol exposed male and female rats. Furthermore, semaglutide reduces the body weight in these alcohol-drinking rats. However, the potential of semaglutide to lower the body weight change is evident after the first injection in the first experiment, while it is demonstrated after the third injection in experiment three. The reasons for this treatment discrepancy are yet to be determined, but may be due to different baseline drinking, different batches of rats or prior HFD exposure. A finding previously shown in preclinical and clinical studies of obesity (Gabery et al., 2020; Wilding et al., 2021; Martins et al., 2022). Collectively, the present findings on feeding and body weight support the contention that GLP-1 signaling controls energy homeostasis. While varenicline neither affect feeding nor body weight, bupropion reduces the food intake in both sexes. A finding in accordance with previous studies [for review see (Gadde and Xiong, 2007)]. As for the outcome on alcohol intake, neither treatment combination additively reduces food intake compared to semaglutide alone. In contrast, bupropion slightly enhances the ability of semaglutide to reduce the body weight of male alcohol drinking rats. These findings provide additional support for combining GLP-1R agonists with other pharmacotherapies for the treatment of obesity (Drucker, 2022). It should however be noted that the treatment effect of body weight was shown as body weight change (experiment 1 and 3) and actual body weight (experiment 2), making the comparison in treatment effect between treatments difficult.

HFD tended to decrease or reduce food intake in the alcohol drinking male and female rats respectively, while the body weight change is unaffected by HFD exposure. Supportively, HFD alters food intake and food-motivated behaviors (Arcego et al., 2020; Altherr et al., 2021). It should however be noted, that the food intake is lower in rats treated with semaglutide and exposed to HFD compared to those only treated with semaglutide. The mechanisms responsible for this potentiated treatment outcome is unknown, and thus a tentative focus for up-coming studies. Intriguingly, HFD tends augments semaglutide’s ability to decrease body weight in male rats, whereas it blunts the same outcome in female rats. The ability of this combination to change body weight in a sex dependent manner may lay in GLP-1R agonists ability to alter neurotransmission divergently (Vallof et al., 2020).

In summary, the present study reveals that semaglutide reduces alcohol drinking, food intake and body weight in male and female rats. Although, the combination of semaglutide with either smoking cessation agents does not add any beneficial alcohol reducing effects, the combination of semaglutide and bupropion may additively reduce body weight in male rats. Consequently, the present study provides further insight into the tentative outcome of different combinatorial pharmacotherapies for the treatment of AUD or obesity.

## Data Availability

The raw data supporting the conclusion of this article will be made available by the authors, without undue reservation.
